# White-nose syndrome detected in bats over an extensive area of Russia

**DOI:** 10.1186/s12917-018-1521-1

**Published:** 2018-06-18

**Authors:** Veronika Kovacova, Jan Zukal, Hana Bandouchova, Alexander D. Botvinkin, Markéta Harazim, Natália Martínková, Oleg L. Orlov, Vladimir Piacek, Alexandra P. Shumkina, Mikhail P. Tiunov, Jiri Pikula

**Affiliations:** 1 Department of Ecology and Diseases of Game, Fish and Bees, University of Veterinary and Pharmaceutical Sciences Brno, Palackého tř. 1946/1, 612 42 Brno, Czech Republic; 2 Institute of Vertebrate Biology of the Czech Academy of Sciences, v.v.i., Květná 8, 603 65 Brno, Czech Republic; 3 Institute of Botany and Zoology, Masaryk University, Kotlářská 267/2, 611 37 Brno, Czech Republic; 4 Irkutsk State Medical University, Krasnogo Vosstania street 1, Irkutsk, Russian Federation 664003; 5 Institute of Biostatistics and Analyses, Masaryk University, Kamenice 126/3, 625 00 Brno, Czech Republic; 6 International Complex Research Laboratory for Study of Climate Change, Land Use and Biodiversity, Tyumen State University, Volodarckogo 6, 625003 Tyumen, Russia; 7 Department of Biochemistry, Ural State Medical University, Repina 3, 620014 Ekaterinburg, Russia; 8 Western Baikal protected areas, Federal State Budgetary Institution “Zapovednoe Pribaikalye”, Baikalskaya st. 291B, 664050 Irkutsk, Russia; 9 Institute of Biology and Soil Science, Far East Branch of the Russian Academy of Sciences, Pr-t 100-letiya Vladivostoka 159, 690022 Vladivostok, Russia

**Keywords:** Chiroptera, Hibernation, *Pseudogymnoascus destructans*, Prevalence, Distribution

## Abstract

**Background:**

Spatiotemporal distribution patterns are important infectious disease epidemiological characteristics that improve our understanding of wild animal population health. The skin infection caused by the fungus *Pseudogymnoascus destructans* emerged as a panzootic disease in bats of the northern hemisphere. However, the infection status of bats over an extensive geographic area of the Russian Federation has remained understudied.

**Results:**

We examined bats at the geographic limits of bat hibernation in the Palearctic temperate zone and found bats with white-nose syndrome (WNS) on the European slopes of the Ural Mountains through the Western Siberian Plain, Central Siberia and on to the Far East. We identified the diagnostic symptoms of WNS based on histopathology in the Northern Ural region at 11° (about 1200 km) higher latitude than the current northern limit in the Nearctic. While body surface temperature differed between regions, bats at all study sites hibernated in very cold conditions averaging 3.6 °C. Each region also differed in *P. destructans* fungal load and the number of UV fluorescent skin lesions indicating skin damage intensity. *Myotis bombinus*, *M. gracilis* and *Murina hilgendorfi* were newly confirmed with histopathological symptoms of WNS. Prevalence of UV-documented WNS ranged between 16 and 76% in species of relevant sample size.

**Conclusions:**

To conclude, the bat pathogen *P. destructans* is widely present in Russian hibernacula but infection remains at low intensity, despite the high exposure rate.

**Electronic supplementary material:**

The online version of this article (10.1186/s12917-018-1521-1) contains supplementary material, which is available to authorized users.

## Background

Any infectious disease determinants associated with the host(s), the agent and the environment will vary geographically [1]. Geographic distribution of infectious diseases is modulated by climate-associated factors inducing changes in the host-pathogen system [2–4]. Variation in the host-pathogen system attributable to climate includes changes in virulence, adaptation of the pathogen to hosts and vectors, the pathogen’s ability to survive in the environment after being shed from the host, along with host population ecology, susceptibility and immune function [5]. Generally speaking, anthropogenic, environmental and ecological factors are drivers of infectious disease emergence [6]. Spatial and temporal distribution data related to infectious diseases are necessary to increase our understanding of population health in wild animals, to identify populations and species at risk, to trace disease origin, to predict and model disease spread and dynamics and to propose effective control measures.

While bats have been recognised as important reservoir hosts for a great variety of emerging infectious agents [7], the fungus *Pseudogymnoascus destructans* [8, 9], causative agent of white-nose syndrome (WNS), is the first pathogen to threaten chiropteran biodiversity [10, 11] in the temperate zone. Constrained by temperature [12] and humidity [13], WNS emerged in a specific niche, i.e. underground bat hibernacula [14]. Breaking out as a point epidemic in eastern North America in 2006 [10], *P. destructans* infection has gradually been recognized as a panzootic in bats of the northern hemisphere [10, 15–21].

Success in WNS surveillance depends on the use of accurate tools and timing of sampling, along with knowledge on the seasonality and natural history of the disease. In addition to qualitative fungus identification using culture and polymerase chain reaction (PCR) [8], quantitative methods such as qPCR [22] and image analysis of photographs taken via trans-illumination of wing membranes with UV light [23] can also be used to evaluate infection intensity [21]. In fact, modification of the Wood’s lamp for UV light diagnostics of WNS is one of the most useful tools allowing immediate recognition of infected bats, the method being highly sensitive and specific in targeting skin lesions for biopsy collection under field conditions. As UV lamp is a non-lethal diagnostic tool allowing rapid examination, it is applicable for the examination of protected bat species. UV transillumination also allows the researcher to distinguish between invasive infection and skin surface colonisation in *P. destructans*-exposed bats [24, 25] as it functions by fluorescing skin lesions laden with vitamin B_2_, that are characteristic of *P. destructans* infection [26].

WNS skin infection has recently been recognised in the West Siberian Plain of Russian Asia [21] and north-eastern China [19]. Widespread endemicity of the WNS fungus in the Palearctic suggests that bat tolerance to this infection probably became established due to long-term co-evolution [21, 27]. Interestingly, presence of the pathogen has also been identified in historic bat populations and the regions of Samara and Irkutsk (European and Asian parts of Russia, respectively) using ethanol-stored samples of bat ectoparasites [28]. Here we further address the infection status of bats over an extensive geographic area of Russia, extending the known northern and eastern geographic limits of the disease and detailing site- and species-dependent differences in epidemiological characteristics.

## Methods

### Bat sampling sites and procedures

**Fig. 1:**
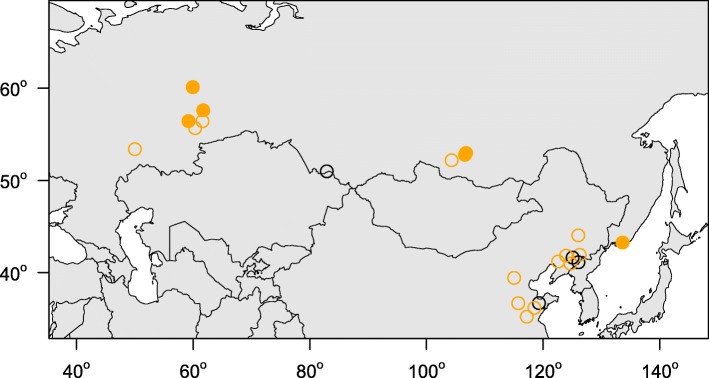
Distribution of study sites in the central and eastern parts of the Russian Federation. Closed circles = this study, open circles = previously published sites [21, 28], orange = *Pseudogymnoascus destructans* infection confirmed with quantitative PCR, black = *P. destructans* not detected

Between 2014 and 2017 we sampled 188 bats (11 species) at 11 hibernation sites from the European slopes of the Ural Mountains through the Western Siberian Plain, Siberia and the Russian Far East (Fig. 1; Table 1). Bats were sampled during the late hibernation season (April and May) and all bats were later released at the capture site. Bat body temperature was measured individually with a Raynger MX2 non-contact IR thermometer (Raytek Corporation, USA) by focusing the laser beam at the central part of bat’s body. Following hand capture, the dorsal side of the left wing was swabbed with a nylon swab (FLOQ Swabs, Copan Flock Technologies srl, Brescia, Italy) for qPCR diagnosis. Presence and quantity of *P. destructans* was assessed using a TaqMan® Universal Master Mix II with UNG (Life Technologies, Foster City, CA, USA) using the dual-probe assay [22]. Optimisation of the PCR reaction and calculation of fungal load was in accordance with Zukal et al. [21] for samples taken between 2014 and 2016 and Zahradníková et al. [28] for samples from 2017. The diagnostic symptoms of WNS were confirmed by current standards. For histopathology analysis, we selected orange-yellow fluorescing spots observed over a 368 nm UV lamp [23]. Suspect wing tissues were biopsied and stored in 10% formalin. The formalin-fixed skin samples were then embedded in paraffin and stained for fungi with periodic acid-Schiff stain. Histological observation took place under an Olympus BX51 light microscope (Olympus Corporation, Tokyo, Japan). Yellow-orange fluorescing WNS lesions on the right wing were manually enumerated on trans-illuminated photographs using the ImageJ counting tool [29].

**Table 1:** Number of bats sampled in Russia

Species	Gender			Total
	Females	Males	NA	
*Eptesicus nilssonii*	9	22		31
*Myotis bombinus*	1	2		3
*Myotis brandtii*	7	11	1	19
*Myotis dasycneme*	30	19		49
*Myotis daubentonii*	1	2		3
*Myotis gracilis*	2	32		34
*Myotis macrodactylus*	1			1
*Myotis petax*	2			2
*Murina hilgendorfi*	19	17		36
*Plecotus auritus*		1		1
*Plecotus ognevi*		8	1	9
Total	80	106	2	188

Gender and species data were obtained for bats included in the study. While each bat was sexed by inspection of external genitalia, species identification was based on morphological traits and/or sequencing the mitochondrial gene for cytochrome b (mtcyb)

### Phylogenetic reconstruction

We sequenced the mitochondrial gene for cytochrome b (mtcyb) in 77 bats in order to validate species identification in newly sampled regions. DNA was isolated from the wing punch biopsies using the DNeasy Blood & Tissue Kit (Qiagen, Halden, Germany), according to the manufacturer’s protocol. We amplified the mtcyb gene with the mammalian primers L7 (ACCAATGACATGAAAAATCATCGTT) and H6 (TCTCCATTTCTGGTTTACAAGAC) [30], supplied at 0.2 μM concentration in a reaction mix containing 1× buffer, 0.2 mM of dNTPs, 2 mM MgCl_2_ and 0.1 U Platinum Taq polymerase (Invitrogen, Life Technologies, Carlsbad, CA, USA). The cycling conditions included a 3 min denaturation step at 95 °C, followed by 36 cycles of 40 s at 95 °C, 50 s at 53 °C and 80s at 72 °C. The reaction was finalised with a 3 min extension at 72 °C. We purified the PCR products through EXO-CIP enzymatic purification and sequenced them commercially with amplification primers from both directions. The chromatograms were assembled in CodonCode Aligner 7.1 (CodonCode, Centerville, MA, USA). Together with mtcyb sequences from GenBank, we aligned the sequences in MAFFT 7.3 [31] and reconstructed the phylogenetic relationships in MrBayes 3.2 [32] using Markov chain Monte Carlo (MCMC) sampling over two independent runs. The MCMC used the HKY + Γ substitution model run for 1 million generations with 30% of initial sampled states discarded as burn in. The settings enabled MCMC convergence and trees in the posterior were summarised with 50% majority-rule consensus.

### Statistical analysis

The common logarithm of *P. destructans* load and the number of UV-documented skin lesions were used for statistical analysis as these variables did not meet normality (Shapiro-Wilk test, *p* < 0.05). Differences between regions and bat species were tested using ANOVA. Body surface temperature could not be transformed to normality; hence, non-parametric Kruskal-Wallis ANOVA was used for the comparison of body surface temperature between regions and bat species. Pearson’s correlation coefficient was used to evaluate the relationship between *P. destructans* load and the number of UV-identified skin lesions. All analyses were performed using Statistica for Windows 12.0 (StatSoft, USA).

## Results

### Locality-dependent differences

**Fig. 2:**
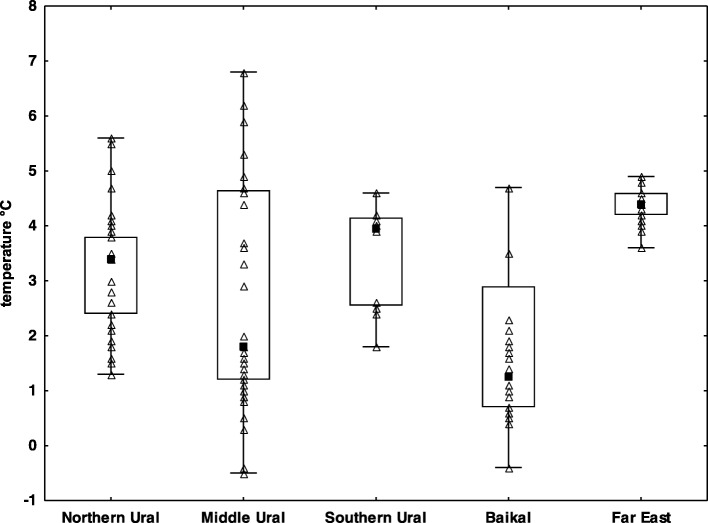
Body temperature of hibernating bats in the study regions. *Explanation*: black square mid-point = median; box = inter-quartile range; whiskers = minimum/maximum range, empty triangles = particular samples

WNS positive bats (both *P. destructans*-positive on qPCR and WNS-positive on UV and histopathology) were confirmed in all study regions (Fig. 1) and at all hibernation sites (Table 2) except the Dachnaya cave, where a single bat was checked with no signs of WNS and negative qPCR results. The sample sites covered the whole of the Asian part of Russia, with additional new sites in the European part. Sites situated in the Northern Ural region were approximately 1200 km further north than any previous site with confirmed WNS in the world. Bats at all study sites hibernated in very cold climatic conditions (median 3.6 °C, min − 0.5 °C, max 6.8 °C), with warmest conditions recorded near the coast at the Primorskiy Velikan cave in the Primorskiy region (Fig. 2). Body surface temperatures differed significantly between regions, with a significant correlation observed between body surface temperature and ambient temperature (Kruskal-Wallis test: *H*_4, 185_ = 39.35, *p* < 0.001). Regions also differed significantly in *P. destructans* fungal load (ANOVA: *F*_4,130_ = 17.11, *p* < 0.001) and number of UV fluorescent skin lesions (ANOVA: *F*_4,66_ = 17.54, *p* = 0.001), with bats hibernating in the Baikal having lowest values of both disease parameters (Table 2).

**Table 2:** Differences in bat communities, hibernation temperature and infection status between sites

Region	Locality	Number of bats	Number of species	WNS UV lesions	WNS qPCR assay	WNS histo-positivity	Median temperature (°C)
Southern Ural	Slyudorudnik mine	12	4	+	+	+	3.95
Middle Ural	Arakaevskaja cave	10	2	+	+	+	1.25
	Smolinskaja cave	21	3	+	+	+	4.70
	Šajtanskaja cave	25	4	+	+	–	1.40
Northern Ural	Dačnaja cave	1	1	–	–	–	3.50
	Komsomolskaja cave	18	3	+	+	+	2.70
	Partizanskaja cave	16	2	+	+	+	3.45
Baikal	Aja cave	2	1	N.A.	+	N.A.	N.A.
	Mečta cave	31	3	+	+	+	1.00
	Cave Vologodskovo	10	1	+	+	+	4.70
Far East	Primorskij Velikan cave	42	5	+	+	+	4.40
*Total number of positive bats*				78	135	48	

Apart from hibernation conditions (body surface temperature of bats), the table includes data concerning bat biodiversity and qualitative measures of *Pseudogymnoascus destructans* infection status

### Differences between species of hibernating bats

**Fig. 3:**
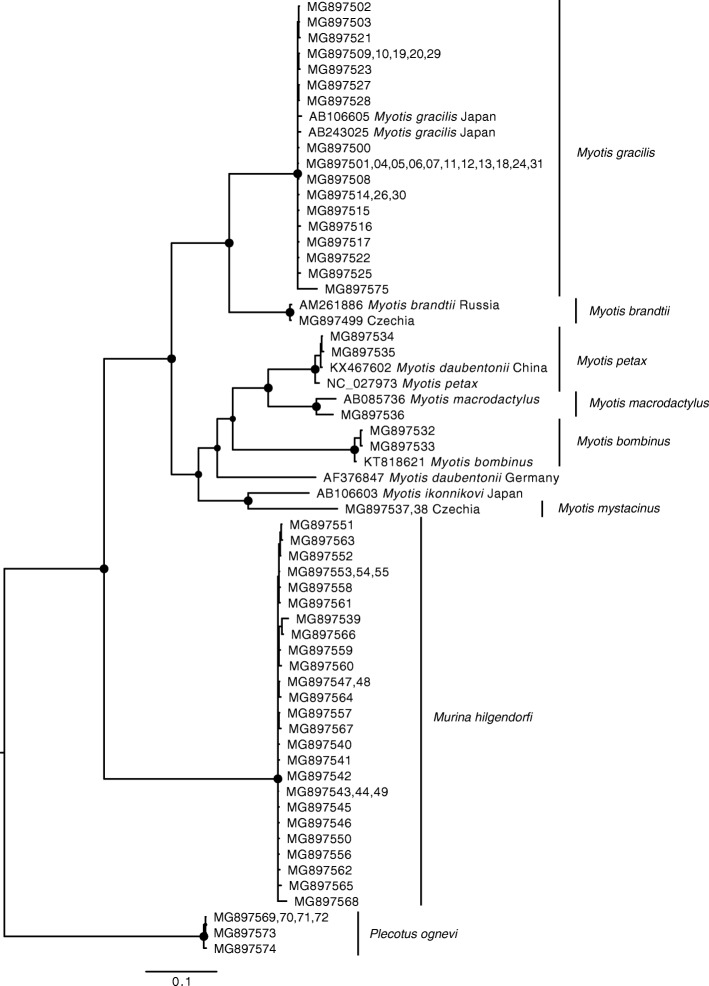
Bayesian inference phylogeny of bats from the Eastern Palearctic based on partial mtcyb gene sequences. Circles at nodes indicate Bayesian posterior probability >0.95; species reported in this study are indicated with vertical bars. Specimen vouchers are listed for newly sequenced individuals, where numbers in curly brackets represent multiple vouchers. Previously published sequences are reported with their accession numbers, species designation in GenBank and country of sample origin

Samples were analysed from 11 bat species covering most of the hibernating bat diversity in the Eastern Palearctic region. Of the 77 bats sequenced (European Nucleotide Archive: MG897500 – MG897575), 25 were identical to others in the dataset. We added nine previously published sequences in order to obtain an alignment containing 60 unique haplotypes of partial mtcyb sequences 1061 bp long. Using Bayesian inference phylogenetic reconstruction (Fig. 3), we confirmed that bat genetic diversity based on the mtcyb gene is consistent with known bat diversity in the Eastern Palearctic.

**Fig. 4:**
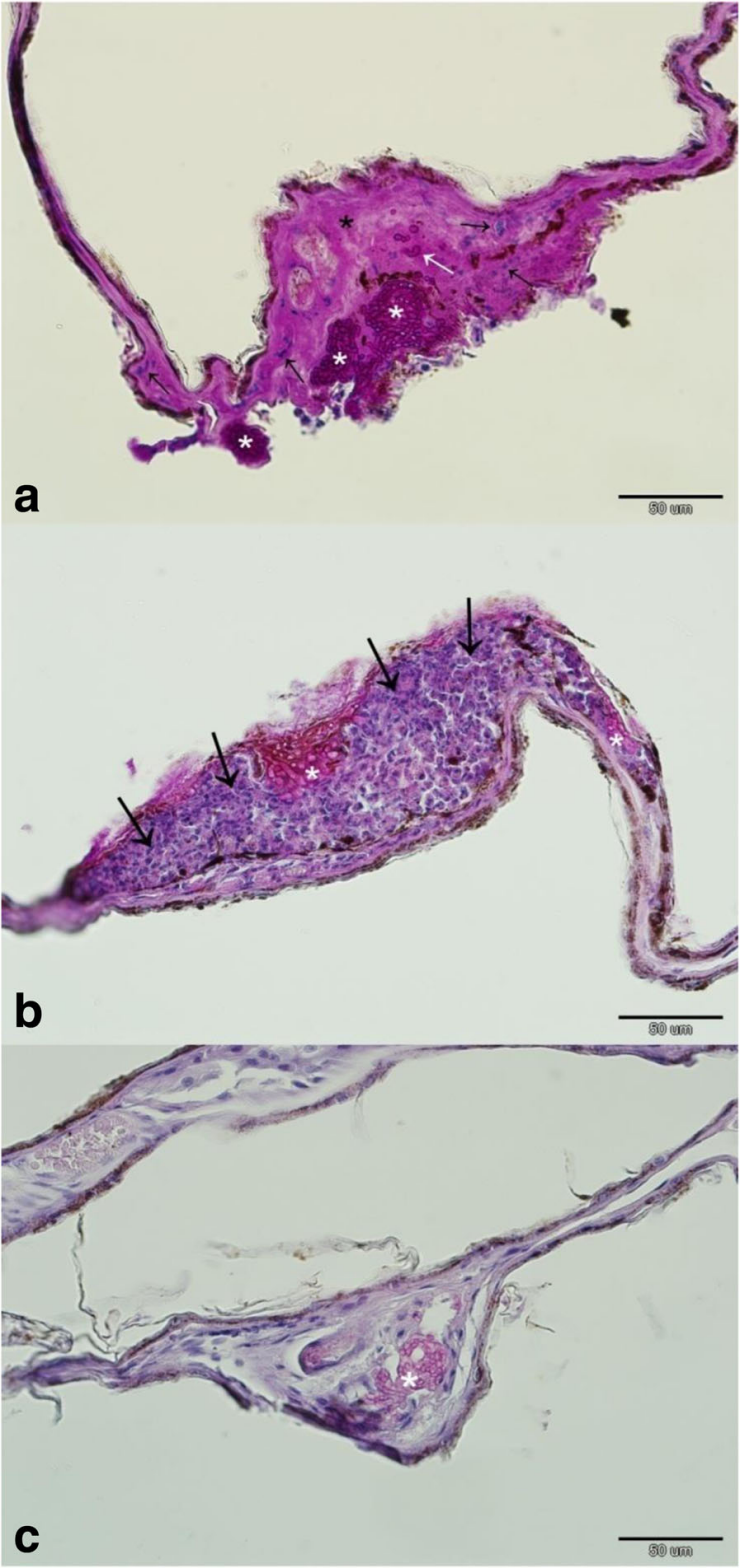
Dermatopathology of *Pseudogymnoascus destructans* infection in Russian bats. **a** *Myotis dasycneme*, Urals: necrotic wing membrane characterised by loss of skin structure and hypereosinophilia (black asterisk), cupping erosions packed with *P. destructans* hyphae (white asterisk) breaching the basement membrane (white arrow), neutrophilic inflammation (black arrow). **b** *Myotis gracilis*, Baikal: fungal cupping erosions (white asterisk) sequestered with neutrophils (black arrow) from the wing membrane. **c** *Murina hilgendorfi*, Primorye: hair follicle (white asterisk) and associated glands infected with the fungus. Periodic acid-Schiff stain

**Fig. 5:**
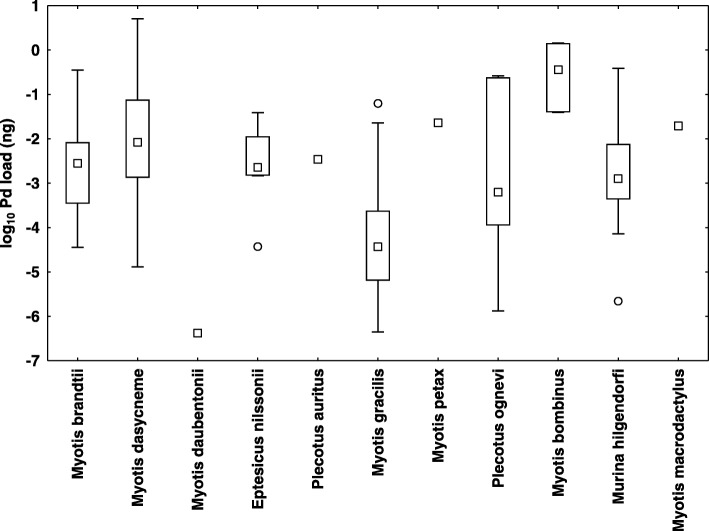
Infection intensity, measured as fungal load in nanograms on a log_10_ scale, for *Pseudogymnoascus destructans* positive bats. *Explanation*: mid-point = median; box = inter-quartile range; whiskers = non-outlier minimum/maximum range; dots = outliers

With the exception of *Myotis macrodactylus* and *Plecotus ognevi*, where only *P. destructans* DNA material was found on the wings, all the bat species monitored were confirmed as both *P. destructans* and WNS positive (Table 3). Three bat species (*M. bombinus*, *M. gracilis* and *Murina hilgendorfi*) are newly confirmed with WNS histopathological symptoms identical with those shown by European and North American bats (Fig. 4). Prevalence of *P. destructans* infection (qPCR) and WNS (expressed as UV fluorescent skin lesions) varied between species (Table 3), with WNS prevalence ranging between 16 and 76% in samples with more than five specimens. Similarly, both WNS impact parameters, i.e. *P. destructans* load (ANOVA: *F*_8,126_ = 9.41, *p* < 0.001; Additional file 1: Figure S1 and Fig. 5) and number of UV fluorescent skin lesions (ANOVA: *F*_7,63_ = 3.32, *p* = 0.005) differed significantly between bat species. There was also a significant correlation between fungal load and number of UV fluorescent skin lesions (*r* = 0.40, *p* < 0.05).

**Table 3:** Prevalence (percentage) with confidence interval of *Pseudogymnoascus destructans* infection in Russian bats

Species	WNS UV-documented skin lesions				WNS qPCR assay				Histo-positivity
	Negative	Positive	Analyzed	Prevalence	Negative	Positive	Analyzed	Prevalence	
*Eptesicus nilssonii*	26	5	31	16.1 ± 12.9	26	5	31	16.1 ± 12.9	+
*Myotis bombinus*	1	1	2	50.0 ± 69.3		3	3	100.0 ± 0.0	+
*Myotis brandtii*	10	9	19	47.4 ± 22.5	7	12	19	63.2 ± 21.7	–
*Myotis dasycneme*	12	37	49	75.5 ± 12.0	7	41	48	85.4 ± 10.0	+
*Myotis daubentonii*	2	1	3	33.3 ± 53.3	2	1	3	33.3 ± 53.3	N.A.
*Myotis gracilis*	26	7	33	21.2 ± 13.9	5	29	34	85.3 ± 11.9	+
*Myotis macrodactylus*	1		1	0.0 ± 0.0		1	1	100.0 ± 0.0	N.A.
*Myotis petax*		2	2	100.0 ± 0.0		1	1	100.0 ± 0.0	+
*Murina hilgendorfi*	21	15	36	41.7 ± 16.1		36	36	100.0 ± 0.0	+
*Plecotus auritus*		1	1	100.0 ± 0.0		1	1	100.0 ± 0.0	+
*Plecotus ognevi*	7		7	0.0 ± 0.0	3	5	8	62.5 ± 33.5	N.A.
Total	106	78	184		50	135	185		8 species

Numbers of positive bats were determined using qualitative characteristics of *P. destructans* infection status examination

## Discussion

Russia’s enormous size and the geographic remoteness of many hibernacula makes active surveillance for bat diseases a difficult task in the Eastern Palearctic. The resulting poor knowledge of bat community infection status over such an extensive understudied territory means that impacts of wildlife conservation concern often go undetected. Further, while single visits to hibernation sites provide static data, they cannot evaluate disease dynamics or changes in bat abundance. However, long-term monitoring of hibernating bats in caves in the study regions have yet to report any mass mortalities or dramatic declines in bat abundance [33–36].

Since 2008, presence of *P. destructans* and/or WNS has been confirmed over an area stretching from Portugal to Turkey [17, 18, 37, 38]. By extending our knowledge on the distribution range of *P. destructans* to the Northern Ural region (forming the boundary between the European and Asian continents) and on to the southern part of the Russian Far East, we come close to covering the geographic limits of bat hibernation in the Palearctic temperate zone [21, 39–41]. In light of current data, the last remaining biogeographic questions regarding WNS distribution in the Palearctic are its presence or absence in Japan, Sakhalin, the Kuril Islands or the Kamchatka Peninsula. Based on its presence in both Continental Europe and the British Isles [42], it is quite likely that *P. destructans* will be found in islands off the mainland of Far Eastern Asia. Furthermore, we were able to confirm histopathological symptoms of WNS (Fig. 4a; [24, 43]) in bats at an 11° (ca. 1200 km) higher latitude than the previous highest finding in the Canadian provinces [44].

In contrast with sites in North America, *P. destructans* in the Palearctic region does not appear to be associated with dramatic bat mortalities [15, 19, 21], despite the number of *P. destructans* or WNS positive bat species being higher in the Palearctic than Nearctic (Additional file 2: Table S1). As previously predicted by Zukal et al. [40], three Asian vespertilionid bat species were newly confirmed with pathognomonic skin lesions induced by *P. destructans* infection in the present study (e.g. Fig. 4b, c). Paired data on identification of *P. destructans* with qPCR and WNS diagnostics on histopathology, supported with host molecular genetic phylogeny from the Eastern Palearctic [45], indicates that WNS affects *M. bombinus*, *M. brandtii*, *M. dasycneme*, *M. gracilis*, *M. petax*, *Eptesicus nilssonii*, *Murina hilgendorfi* and *Plecotus auritus* in Russia. Both *M. daubentonii* and *M. macrodactylus* were found to be infected with *P. destructans*, though WNS has not yet been confirmed on histopathology. Russian Siberian bats invaded by the fungus show clearly visible orange–yellow fluorescence of wing membrane skin lesions under UV light, documenting that this diagnostic tool [23] is applicable throughout the known distribution range of the pathogen and, moreover, that Russian *P. destructans* strains hyperproduce vitamin B_2_, a WNS virulence factor [26].

The WNS fungus is a generalist pathogen of hibernating vespertilionid and rhinolophid bats [40]. Assuming that all bats (with species-specific behavioural and roosting variation) that enter a *P. destructans*-contaminated hibernaculum have an equal chance of exposure to the pathogen, prevalence (percentage of bats positive for the agent) documented in Russian bats should then be an indicator of a high environmental contamination level and exposure rate. In this study, fungal load, a function of variables such as the infectious dose, duration of infection and growth rate of the agent in given environmental conditions, showed site- and species-dependent differences. Bat hibernation in the Urals, Siberia and the Russian Far East lasts up to 7 months and is confined to underground shelters due to strong frosts during winter. Temperatures in such hibernacula do not exceed 5 °C throughout the year [33–36]; hence, Russian bats should show lower fungal loads than European and North American bats as they hibernate under colder microclimatic conditions, which lead to slow temperature-dependent growth of the pathogen [12]. The prevalence of UV-documented skin lesions and histopathological positivity in this study signifies species susceptibility to infection. As the probability of serious wing membranes damage increases with increasing fungal load [25], and a lowered fungal load is linked with lower WNS impact (expressed as reduced UV fluorescent lesions, similar to [21]), we suggest that bats hibernating in such cold climatic conditions have a higher probability of surviving infection than elsewhere [46].

## Conclusion

While it is not known how long the *P. destructans* fungal pathogen has been present in the Palearctic region, or whether there were periods of mass mortality associated with infection in the past (cf. [15]), our data suggest that its geographic expansion apparently covers the whole Palearctic niche of bat hibernation. The circumstances that influence its potential to cause morbidity and/or mortality in bats in Palearctic Russia are poorly understood; however, *P. destructans* is a significant disease-causing pathogen of hibernating bats, discovery of which warrants development of active surveillance programmes to better understand its epizootiology and to protect wildlife in general. This programme could be combined with testing for other agents of zoonotic importance, such as rabies.

## Additional files

Additional file 1: **Figure S1.** Infection intensity, measured as fungal load in nanograms on a log_10_ scale, for particular regions. *Explanation*: mid-point = median; box = inter-quartile range; whiskers = non-outlier minimum/maximum range; dots = outliers; stars = extremes. (DOCX 21 kb)

Additional file 2: **Table S1.** Bat species from Palearctic and Nearctic regions with confirmed WNS or *Pseudogymnoascus destructans* infection. Summarized from [10, 16, 19, 21, 40, 47]. (XLSX 13 kb)

Additional file 3: **Table S2.** Datasets used and analyzed during the current study and not included in Tables 1, 2 and 3. (XLSX 31 kb)

## Abbreviations

MCMC: Markov chain Monte Carlo
UV: Ultraviolet
WNS: White-nose syndrome
