# Supplementary material for: White-nose syndrome detected in bats over an extensive area of Russia
Source: BMC Vet Res. 2018 Jun 18;14:192. doi: 10.1186/s12917-018-1521-1 (PMC6007069; doi:10.1186/s12917-018-1521-1)
Supplement: Supplementary file 1 — Figure S1. Infection intensity, measured as fungal load in nanograms on a log10 scale, for particular regions. Explanation: mid-point = median; box = inter-quartile range; whiskers = non-outlier minimum/maximum range; dots = outliers; stars = extremes. (DOCX 21 kb) [file 12917_2018_1521_MOESM1_ESM.docx]

**Additional file 1: Figure S1.** Infection intensity, measured as fungal load in nanograms on a log_10_ scale, for particular regions. *Explanation*: mid-point = median; box = inter-quartile range; whiskers = non-outlier minimum/maximum range; dots = outliers; stars = extremes.
